# Leadership Ostracism Behaviors From the Target’s Perspective: A Content and Behavioral Typology Model Derived From Interviews With Chinese Employees

**DOI:** 10.3389/fpsyg.2019.01197

**Published:** 2019-05-24

**Authors:** Mengchu Zhao, Zhixia Chen, Mats Glambek, Ståle V. Einarsen

**Affiliations:** ^1^Department of Public Administration, College of Public Administration, Huazhong University of Science and Technology, Wuhan, China; ^2^Department of Psychosocial Science, Faculty of Psychology, University of Bergen, Bergen, Norway

**Keywords:** leadership ostracism, behavioral typology model, in-depth interview, grounded theory, China

## Abstract

Leadership ostracism denotes a severe work stressor, potentially entailing more serious negative effects than other types of workplace ostracism. However, scholars have paid relatively little attention to ostracism carried out by leaders, leaving the phenomenon insufficiently accounted for in the literature. Hence, the present study aims to explore the content and typology of leadership ostracism behavior by in-depth interviews and inductive analyses based on grounded theory, in order to give a thorough presentation and description of the leadership ostracism concept as perceived and construed by Chinese subordinates. Respondents were invited using a snowball sampling technique, and the final sample consisted of 26 individuals employed in different Chinese firms. Based on the reported experience of the interviewees, 11 concrete leadership ostracism behaviors emerged from the data. Further analyses revealed a leadership ostracism behavioral typology model reflecting five core categories, i.e., *general ignoring, neglect, exclusion, differential treatment*, and *undermining*. These findings appear to partly replicate and partly expand on previous conceptualizations of workplace ostracism, indicating that leadership ostracism may reflect a distinct variant of the phenomenon, eligible to be studied in its own right. The present study also discusses certain culture-specific aspects of leadership ostracism that can be taken into consideration in future studies.

## Introduction

According to social psychologists, individuals have a strong desire to establish and maintain secure and positive social group memberships, and are driven by a fundamental need to belong (Baumeister and Tice, 1990; Strauss and Corbin, 1990). Drawing on tenets of evolutionary psychology, such a need is often explained in terms of the protection and reproduction opportunities represented by group membership, and is therefore likely to have evolved through natural selection, not only in humans, but also among other social species (Gruter and Masters, 1986; Williams, 2001, 2007). As a result, fear of exclusion from important social groups and relationships is likely to be deeply rooted in humans, as is the inclination to respond with anxiety and pain when such exclusion is imminent and ongoing (Eisenberger et al., 2003). This notion has spurred a growing research interest with respect to the phenomenon of ostracism, i.e., social interactions in which one or more group-members are ignored and excluded, be it by a significant other individual or by a relevant group (Williams, 2007). In fact, numerous examinations of ostracism and its effects have been carried out across different research designs in recent years, from laboratory experiments (e.g., Stroud et al., 2000; Eisenberger et al., 2003) to qualitative analyses (e.g., Williams et al., 2000; Zadro et al., 2004; Zadro and Williams, 2006), with most studies concluding that ostracism is both prevalent and harmful (e.g., Williams, 1997, 2001, 2007; Eisenberger et al., 2003; Gunnar et al., 2003).

As part of this initiative, research on ostracism has also been extended to organizational contexts. Organizations represent a unique social context in this regard, as individuals are bound to them not only through social attachment, but also by economic and practical incentives. Thus, in many cases, ostracism within organizations may be particularly harmful, as the victims cannot simply leave and easily seek inclusion elsewhere. In addition, psychological responses to perceived ostracism may negatively affect organizational outcomes. For example, in an interview study among employees who felt ostracized in the workplace, Williams (2001) found that the experience increased the targets’ inclination to respond with anxiety and pain, as well as to call in sick or to leave organization altogether.

Still, in spite of significant efforts to document ostracism in organizations, one particular version of ostracism in the workplace has yet to be adequately illuminated, namely that of leadership ostracism (also called supervisor/supervision ostracism), i.e., being subjected to ostracism behaviors carried out by one’s immediate supervisor. Specifically, although leaders’ positional power has been suggested to prolong the distress of ostracism (Nezlek et al., 2012), and possibly to yield different effects on employees’ work-related behaviors (e.g., Hitlan et al., 2006; Jones et al., 2009; Jahanzeb et al., 2018), few, if any scholars, have attempted to explore the content and typology of leadership ostracism from the subjective viewpoint of the targets.

In the present study, we put the concept of leadership ostracism in the forefront of our investigation, seeking to explore and describe the nature of this particular form of ostracism based on in-depth interviews with subordinates. Specifically, we will look at leadership ostracism in a Chinese cultural context, where the relationship-oriented social structure at work is especially recognizable, making it an ideal setting for our explorative research. The study will hence entail significant contributions to the literature, potentially with important theoretical and practical implications within research on leadership, ostracism and employee well-being.

## The Nature, Sources and Outcomes of Ostracism

The experience of feeling invisible, of being excluded and rejected from the social interactions of those around, of being treated as though one did not belong with the others or even exist, has been demonstrated as a pervasive phenomenon across a broad range of social contexts (Williams, 2001). Such experiences are often collected under the term ostracism (Williams, 1997, 2007), but are also reflected in a number of related words and phrases. In everyday language, terms such as “shunning,” “avoiding,” “estrangement,” “exiling,” “expulsion,” “banishment,” “ignoring,” “giving someone the silent treatment,” “freezing someone out” and “giving the cold shoulder” are examples of acts and experiences likely to reflect variants and manifestations of the ostracism phenomenon (Williams, 1997, 2001). However, this diversity in terms is also evident in the scholarly literature. For example, *shunning* specifically refers to “the deliberate and systematic exclusion of an individual who was once an included member of the group” (Anderson, 2009). *Rejection* has been used to refer to an explicit declaration that one is refused when seeking to form and maintain at least a temporary alliance or relationship with a group or an individual (Leary, 2005; Blackhart et al., 2009), while *social exclusion* denotes situations where the target is denied valued social contact with others (Twenge et al., 2001). Importantly, these constructs seem to be rooted in the same underlying phenomenon, and are often used interchangeably to refer to “a general process of social rejection or exclusion” (Gruter and Masters, 1986). Although semantic and psychologically meaningful distinctions among these constructs are apparent (Williams, 2007), such specific behaviors as exclusion, shunning, ignoring and rejecting share the core characteristic that they all involve omission of socially appropriate behavior, resulting in a feeling of not being included, acknowledged or accepted among those targeted (Robinson et al., 2013). We therefore collectively place such experiences under a broader construct labeled ostracism, which can be characterized by both acts of omission as well as open acts of social exclusion.

The term ostracism itself dates back to the fifth century B.C., originating from the Greek *ostrskismos*, which refers to a practice in ancient Greece where one voted to expel former leaders with dictatorial ambitions from the democratic state as a form of punishment (Zippelius, 1986; Williams, 1997). Specifically, citizens would write the names of such individuals on *ostraca*, pieces of broken clay pottery, with those receiving the most votes subsequently sentenced to years of solitude in exile. The very act of excluding unwelcome and unwanted individuals from the social fellowship or the group is, however, universal. For instance, Williams (2001) noted that different forms of ostracism have been observed in human interactions among tribespeople throughout the world, among individuals or groups across a range of institutions, among children in school, in intimate relationships as well as among several animal species, such as primates, lions and wolves (see Gruter and Masters, 1986; Williams, 1997, 2001, 2007). The universality of ostracism itself indicates that ostracizing certain individuals may serve adaptive purposes in natural selection, implying that the phenomenon is ubiquitous and powerful (Williams, 2001). Specifically, as the evolution of social animals such as humans have been dependent on tight cooperation in relatively small groups and societies for survival and reproduction, the ability to exclude members who fail to contribute to or obscure the functioning of the group is likely to be naturally selected. Moreover, even when ostracism does not take the form of total exclusion from the group, behaviors such as ignoring, rejection and threats of exclusion may serve as correctives or warnings to group members who fail to contribute to or otherwise hinder the group’s functioning.

Similarly, common reactions to ostracism can also be understood in light of evolutionary mechanisms. Scholars suggest that inclusion in social groups has been essential for survival during the human phylogeny, and that the distress and anxiety associated with being ignored and excluded comprise a naturally selected reaction developed to motivate individuals to seek re-inclusion or attempt to remain included (Weir, 2012; Wesselmann et al., 2012). Thus, although ostracism no longer represents a direct survival threat in modern life, the automatic responses it causes are possibly rooted in the fear of death itself, which makes it a particularly distressing form of mistreatment (Giorgi et al., 2016). In order to account for this, Williams (1997, 2001) has proposed a need-threat model of ostracism, positing that ostracism thwarts the fundamental needs of belongingness, perceived control, self-esteem and meaningfulness in life. Williams et al. (2000) has also shown that when ostracized, individuals are more likely to display self-doubt, emotional distress, anxiety, and anger. Reactions to ostracism include acts of aggression (Twenge et al., 2001), self-defeating behaviors (Twenge et al., 2002) and a variety of behaviors related to problems with self-regulation (Baumeister et al., 2005).

Moreover, for the same reason, ostracism hurts in spite of what the perpetrators’ intentions or motives may be. In some cases, ostracism is used intentionally to punish, retaliate or hurt targets, or to avoid interactions with targets for the sake of protecting oneself, be it to avoid conflicts, social awkwardness, or unpleasant emotions. Non-purposeful ostracism without any noxious or harmful intention, however, such as “non-behavior” where the target expects a response or action that does not take place, may be as common and harmful as intended ostracism (Williams, 2001). As a result, acts of ostracism can be quite ambiguous not only regarding why they took place, but also with respect to whether they occurred at all (Robinson et al., 2013).

## Workplace and Leadership Ostracism

As outlined, ostracism is a subtle, yet universal and highly distressing form of interpersonal mistreatment (Lustenberger and Williams, 2009). Its inherent ambiguity and the essential feature of being denied social connection are likely to fuel negative consequences for victims both psychologically and possibly physically (Riva et al., 2011). Still, the way in which ostracism is enacted and impacts employees in organizational settings has rarely been studied, a fact that holds especially true for ostracism carried out by one’s immediate leader.

*Workplace ostracism* has been defined as “the exclusion, rejection, or ignoring of an individual (or group) by another individual (or group) that hinders one’s ability to establish or maintain positive interpersonal relationships, work-related success, or favorable reputation within one’s place of work” (Hitlan et al., 2006, p. 217). Alternatively, workplace ostracism can be defined as omissions to “take actions that engage another organizational member when it is socially appropriate to do so” (Robinson et al., 2013, p. 206). Thus, ostracism in an organizational context involves behaviors that in effect disconnects an employee from valued interpersonal relations at work, for example from co-workers, superiors or the social fellowship at large, yet without necessarily involving an active and formal breach of the employment contract. It may in contrast hinder task completion and efficient work-related collaboration, thereby deterring individual and organizational efficiency.

Interestingly, since the introduction of the concept of workplace ostracism, it has commonly been assumed to constitute a general phenomenon, without any specific reference to its source (Hitlan et al., 2006). However, based on the need-threat/need fortification framework of Williams (2007), different sources of workplace ostracism (e.g., coworker, leader, or entire work groups or organizations, see Williams, 2001) may possibly trigger different psychological processes (Wu et al., 2016; Jahanzeb et al., 2018), implying that it may be worthwhile accounting for the source of the behavior. A study by Jahanzeb et al. (2018), for instance, showed that ostracism from supervisors primarily threatens victims’ efficacy needs, which then leads to defensive silence and emotional exhaustion. Ostracism from co-workers, on the other hand, may trigger threats to relational needs, such as self-esteem and belongingness (Balliet and Ferris, 2013). In another study, Hitlan and Noel (2009) showed that ostracism from colleagues was related to person-targeted counter-productive behavioral responses from the recipient, while ostracism carried out by leaders was associated with counter-productive behavior targeted at the organization. Thus, although research on different sources of organizational ostracism is scarce, there are some evidence suggesting that leadership ostracism can yield distinctive outcomes in this regard.

From a theoretical viewpoint, several different explanations for this tendency are plausible. For instance, via the formal power inherent in their leader position, leaders are highly influential with respect to the social work environment and may affect the employees’ professional reputation and status, their inclusion in social and professional activities and even their possibility to be physically present. Hence, the hierarchical discrepancy between a leader and his/her subordinates may facilitate a set of ostracism behaviors that do not fully overlap with other types of workplace ostracism. In terms of the subjective experience itself, being ostracized by a leader may also give rise to different attributional processes than ostracism enacted by co-workers. Subordinate targets may for example interpret otherwise legitimate behaviors as ostracism precisely because someone in an authority position is enacting them, such as lack of expected support or interpersonal recognition. Hence, subordinates may also be more astute at identifying ostracism from superiors than similar behavior stemming from other organizational members (Spoor and Williams, 2007), having a particular need of recognition, acknowledgment and support from their leader, with treatment from those in authority positions playing a significant role in their work-related self-evaluation (Lind et al., 1998). For instance, subordinates may interpret leaders’ behavior as a reflection of their own social status and standing in the organization, possibly with implications for perceptions of the degree of supervisory support and resources available to them (Lee and Tiedens, 2001).

Together, the theoretical rationale outlined here and the scattered empirical evidence available (e.g., Wu et al., 2012; Wang and Hsieh, 2013) suggests that in terms of the subjective experience, leadership ostracism may not be identical to other forms of workplace ostracism, but may vary both in terms of the behaviors involved and the subjective interpretation of those behaviors. As of yet, however, leadership ostracism does not seem to have been explored using inductive methodology, and the question of how leadership ostracism emerges as a phenomenon from the subjective viewpoint of subordinates remains unanswered.

## The Present Research Context

As Robinson et al. (2013) notes, ostracism is partly characterized by behavior not considered socially appropriate for the context where it occurs. Thus, ostracism takes place when actors fail to provide interpersonal acknowledgment, responsiveness and inclusion in a social context where such behaviors are expected and in line with prevailing social norms.

Chinese cultural values tend to emphasize high power distance and a general unquestioning respect for authority (Ho and Chiu, 1994; Farh et al., 1997). In China, leaders are often given the power of dominance over organizational resources, and possess authority and high organizational status, providing a particularly potent context for studying how ostracism is enacted and how it influences recipients (Chen and Tu, 2017). In addition, the Chinese generally have a deep-rooted desire to pursue moderation and harmony, and are especially devoted to maintain “face” and reputation. Hence, Chinese leaders may be particularly inclined to prefer covert strategies to uphold their authority, avoiding unnecessary direct conflicts.

Related to this, social interactions in China are influenced by the traditional value orientations of “guanxi.” This refers to “an intricate and pervasive relational network to secure favors in personal and organizational relations” (Park and Luo, 2001, p. 455). Moreover, as postulated by the Chinese scholar Fei (1947/1992), interpersonal relationships and social networks in Chinese society are themselves structured in accordance with “guanxi,” in a manner that can be metaphorically depicted as circles in the water. Different circles correspond to different layers in one’s personal social network, with the smaller circles toward the center denoting closer relationships. With respect to leader-subordinate relationships, leader “guanxi” thus entails a form of social categorization, where subordinates are ordered in different circles according to the intimacy, quality and importance of the given relationship (Luo et al., 2016). Consequently, organizational resources and emotional support are distributed in an unbalanced manner subject to the leader’s personal preferences and value judgment.

The “guanxi” structure may itself be unproblematic, as it is often agreed-upon by those involved as a form of unspoken psychological contract (i.e., it complies with prevailing norms). However, when leaders treat subordinates as if they belong to more distal circles than that stipulated in this psychological contract, “guanxi” may represent a potential base for leadership ostracism. As such, leadership ostracism behavior may, at least in China, comprise behavior over and above “ignoring and excluding others in one’s presence” (Williams, 2007). Together, we believe that these culturally specific conditions make the Chinese labor market a well-suited research context for the present study, where ostracism behaviors as carried out by a leader are likely to be highly noticeable and significant from the recipient’s perspective, possibly including a wide array of more or less subtle behaviors.

As leadership ostracism research is scarce in the literature, even though the leaders’ status in the work group entails particular opportunities and possibilities for carrying out such behavior against subordinates, the main aim of the study is to explore the nature of ostracism as carried out by a leader toward his or her subordinates. Specifically, using in-depth interviews, we will attempt to establish a content and behavioral typology model for leadership ostracism as it emerges from the reported experience of Chinese workers.

## Methodology

### Data Collection and Sample

As research on leadership ostracism is yet a novel endeavor, we employ a qualitative interviewing technique to unveil the nature of leadership ostracism behaviors in a Chinese context, as perceived and construed by subordinates. Unlike the large-scale random sampling of quantitative research, qualitative methodology place greater value on the appropriateness of the sample and the richness of data sources (Bazeley, 2004). Thus, the depth and breadth of interview data should be appropriate (Kelley et al., 2003). In order to allow for representativeness of Chinese workers, we placed no constraints on participants’ age, gender, educational background, occupation, average working tenure or full-time/part-time employment status. We thus used a cross-sectional qualitative sampling method, which involves sampling from diverse backgrounds and occupations in an effort to obtain broad ranging data (Bryman, 2004). Generally, the number of interviews should be limited to that of so-called theoretical saturation, i.e., the point where additional interviews fail to add new and distinct variance to the coding categories created by previous interviews (Strauss and Corbin, 1998). In the present study, we found that saturation was reached at 26 interviews, in line with similar studies on ostracism (e.g., Waldeck et al., 2015) and other forms of workplace mistreatment, such as bullying (e.g., Strandmark and Hallberg, 2007). In-depth, semi-structured open-ended interviews were carried out with each respondent.

At the first sampling stage, 23 respondents were contacted either by phone or by email using a snowball sampling technique (i.e., a small sample was recruited through an internet recruitment notice, before additional participants were invited using the social networks of the initial respondents), being invited to take part in an interview regarding the nature of their interaction with their respective superior. Respondents who did not report frequent interaction with their immediate leader or who were the only subordinate under a given leader were excluded from the initial sample (*N* = 3). Subsequently, in a second sampling stage we added six respondents with at least one co-worker in the same position as them. Participants represented a variety of public and private sector organizations including public universities, large state-owned firms as well as private firms. Among the participants, 11 (42.3%) were male and 15 (57.7%) were female. Fourteen respondents were aged 27–30 (53.9%), nine were aged 31–40 (34.6%) and three were under 27 years of age (11.5%). Their educational background ranged from bachelor to doctoral degree, and tenure ranged from 1 to 10 years in the current organization. Respondents were employed in different kinds of institutions, including public institutions (*N* = 5, 19.2%), for example a lecturer at a university; large state-owned firms (*N* = 8, 30.8%), for example a technician in a petrol firm; and private firms (*N* = 13, 50%), for example a sales assistant in an insurance company.

Interviews were carried out in Chinese, either in person (*N* = 4) or by telephone (*N* = 22), and lasted from 30 min and up to 1 h in length, in accordance with similar studies (e.g., Testa and Sipe, 2012; Wilhelmy et al., 2016). Furthermore, a structured protocol with seven main questions (see Appendix) was employed. Given that descriptions of leaders’ behavior by subordinates can yield sensitive information, particularly in a Chinese context, we contacted the participants prior to the interviews to explain the main purpose of the study with assurances of strict confidentiality. Interviews were recorded, and subsequently transcribed using the software Ifyrec before the analyses commenced.

As we aimed to explore the phenomenon of leadership ostracism as experienced or witnessed by the respondents, interviewers employed open questions concerning leadership ostracism, e.g., “could you talk about how you understood leadership ostracism when you first heard about it? It is a term describing the phenomenon where a leader ostracizes his or her subordinates.” Interviewers used the Chinese word “
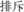
(pái chì),” which translates into “ostracize” both as a verb and as a noun, and is a well-known term in daily language. In some cases, follow-up questions were used in order to facilitate more deliberation about relevant experiences, e.g., “have you ever heard that colleagues or friends say they have experienced leadership ostracism? Please give some examples.”

After completion of all interviews, the information was analyzed in accordance with principles from grounded theory (Glaser and Strauss, 1967; Charmaz, 2000), in order to document the behaviors associated with leadership ostracism from a target perspective. Throughout these analyses, we made sure to keep a broad understanding of the ostracism concept in mind, thus ensuring that even uncommon ostracism behaviors were included while behaviors clearly reflecting other types of mistreatment unrelated to ostracism, such as bullying (see Einarsen et al., 2003) or abusive supervision (see Tepper, 2000), were excluded from further analyses.

### Ethics Statement

An ethics board approval was not required as per institutional guidelines and national laws and regulations since this research did not involve human clinical trials or animal experiments. However, the research was conducted within ethical guidelines. Respondents were informed about the goal of the study for scientific research and gave written informed consent in accordance with the Declaration of Helsinki, and were ensured of confidentiality and anonymity.

### Coding and Analysis Strategy

Following the principles of grounded theory, inductive analysis allows the researcher to continually create and refine categories in an effort to develop a theory that explains a certain phenomenon (Katz, 2015), in alignment with the goal of the present study. Such a process entails a series of separate but related coding and categorization operations, through which major themes are discovered “through the analysts’ interactions with the data” (Patton, 2005, p. 453). In the present study, the categorization process was carried out in two rounds, with several researchers representing two different cultural backgrounds (i.e., Chinese and Norwegian) involved.

The goal of the first step was to break the data into conceptual components, in which useful concepts identified from the data were refined and conceptualized as first-order codes (cf. Strauss and Corbin, 1994). This step involved thorough content analyzing, in which key phrases related to leaders’ ostracism behavior were screened and marked line by line, thus ensuring a range of summary labels that could sufficiently account for themes across the data. This was carried out by three analysts (Ph.D.-students from China), who read and re-read the interview transcript to ensure that all relevant key information was detected and coded. As the analysists’ observations and subjective understanding of leadership ostracism behavior inevitably influences such a process, we adopted the respondents’ own words as labels for the first-order codes. In the case that the same behavior was reported in different ways by different respondents, the phrase or description reflecting the underlying behavior most precisely was chosen, while synonymous phrases were counted as examples of that particular phrase. For example, phrases like “never says a hello to me” and “say nothing when passing by me” were identified and considered as expressing the same meaning and were summarized under the label “(1a) not greeting me.”

In the second step, the analysis took the form of a two-stage categorization process where the research team met at each phase of the data reduction process to assign subclass-units into higher-level classes until consensus was established (cf. Charmaz, 2008). We borrowed a typical coding paradigm involving “conditions, context, action/interactional strategies and consequences” to link relevant code units with specific categories, giving each category a name at the same time as new, larger concepts were generated (Strauss and Corbin, 1990, p. 96).

More specifically, in the first categorization stage, we identified subcategories involving larger, more inclusive concepts or themes that emerged naturally from preliminary first-order summary labels, comparing differences and similarities to see whether they led to the same categories (Sbaraini et al., 2011). Furthermore, we remained attentive to how these abstract concepts were related to existing terms and concepts and how existing research and theory could be used to identify and name new categories (Locke, 2000). Thus, words such as “ignoring” and “excluding” which are characteristic of ostracism in extant literature were adopted for the purpose of naming sub-categories, as the aim of the present research is to expand on existing theories (Shah and Corley, 2006). Any subcategories and category changes put forward by the analysists were documented for further discussion.

The second–stage categorization focused mainly on how the core categories emerged from the lower-level concepts (Glaser and Strauss, 1967). Specifically, in order to move from a mere descriptive level to a conceptual level, the main purpose of this step was to identify any internal relation between the subcategories in order to group them into higher order dimensions (i.e., core categories). Subsequently, analysts with a different cultural background repeated the entire coding and categorization process in order to ensure interrater reliability and to reduce any cultural bias in the coding process. Next, in a joint meeting, each of the analysts presented their own ideas about the coding categories and code definitions compared with that of the other analysts. Comparative analyses with respect to disagreements on the classification of categories and naming of subcategories were continued until agreement was reached, including the core categories, subcategories and first-order summary labels.

## Results

**FIGURE 1:**
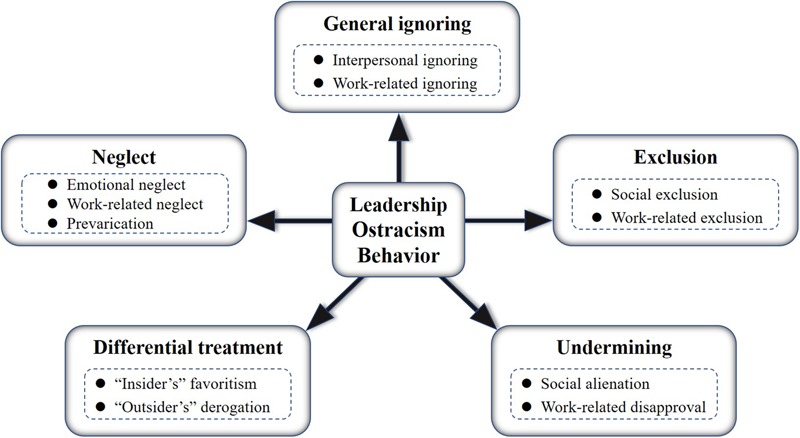
A behavioral typology model of leadership ostracism.

After reviewing the interview transcripts, a total of 297 key phrases were identified. Based on these, 56 first-order behavioral labels were generated (see Table 1), reflecting specific ostracism acts. Moreover, through the secondary analyses, these labels were divided into 11 categories, which could be further grouped together under five core categories, each reflecting a distinct facet of leadership ostracism (see Figure 1). Specifically, the core-categories identified were; *general ignoring* (comprising the sub-categories interactional ignoring and work-related ignoring), *neglect* (comprising the sub-categories emotional neglect, work-related neglect and prevarication), *exclusion* (comprising the sub-categories social exclusion and work-related exclusion), *differential treatment* (comprising the sub-categories “insider’s” favoritism and “outsider’s” derogation) and *undermining* (comprising the sub-categories work-related disapproval and social alienation). For an overview of all sub-categories and their descriptions, see Table 2.

**Table 1:** Categories and behaviors of leadership ostracism resulting from interviews.

Core categories	Subcategories	Conceptual labels (abstracted from marked respondents’ statements)
Ignoring	Interpersonal ignoring	(1a) not greeting me
		(1b) not responding to my warm greeting
		(1c) stopping talking or walking away immediately because of my approach
		(1d) rarely or only briefly communicating with me
		(1e) ignoring my existence at work; treating me as if I am invisible
		(1f) not responding when I express opinions
		(1g) not replying to my messages or e-mails
		(1h) showing an impatient attitude and wanting to finish our conversation quickly
		(1i) conveying work-related information through others
		(1j) not taking the initiative to talk to me compared with my colleagues
		(1k) ignoring my contributions to the group
	Work-related ignoring	(2a) giving me the cold shoulder even when I show great progress at work
		(2b) passing me by when everyone has a chance to express opinions in meetings or discussions
Neglect	Emotional neglect	(3a) not proactively coming to know my difficulties at work compared with other colleagues
		(3b) not comforting me when I feel discouraged encountering obstacles or difficulties
		(3c) not complimenting me when I need encouragement
	Work-related neglect	(4a) not adopting my ideas and suggestions even when my colleagues regard them as reasonable
		(4b) not introducing me when I should be introduced
		(4c) not taking into consideration my requirements (needs) when making decisions relating to my job
		(4d) concealing important information related to my job from me
		(4e) putting aside any complaints or problems I report to senior leaders
		(4f) unwillingness to use his/her power to help me solve problems I encounter at work
		(4g) unwillingness to deploy manpower to help me get out of from a short-handed situation
		(4h) unwillingness to assign me important tasks even if I am competent for them
		(4i) providing me with less opportunities for training even though I am well qualified
		(4j) not recommending me for raises, awards or promotions even if I perform excellent at work
	Prevarication	(5a) unwillingness to make any explanations when I have a complaint about decisions involving me
		(5b) responding indifferently when I consult him/her
		(5c) finding some excuse to refuse me when I ask for help
Exclusion	Social exclusion	(6a) not informing me to join a dinner organized by the team or the department
		(6b) not inviting me to attend collective activities
		(6c) inviting other colleagues to join his/ her after-work gathering but not me
	Work-related exclusion	(7a) not bringing work-related activities to my attention (like important meetings or business trips)
Differential treatment	“Insider’s” favoritism	(8a) giving other colleagues priority when there are good opportunities
		(8b) choosing to trust other colleagues before me
		(8c) preferring my colleagues over me for certain positions that can generate immediate benefits
		(8d) sharing ideas with my colleagues, but not with me
		(8e) taking a friendlier tone of conversation with other colleagues than with me
		(8f) giving my colleagues the opportunity to choose job tasks before me
	“Outsider’s” derogation	(9a) being angry with me for making jokes about him/her, but not with other colleagues
		(9b) sacrificing my interests to meet the needs of other colleagues
		(9c) making it more difficult for me to get firsthand information
		(9d) giving me less opportunity to express my opinions compared with my colleagues
		(9e) giving me lower performance rates than others who perform similar to me
		(9f) providing me with less convenience at work than other colleagues
		(9g) providing me with less resources than other colleagues
		(9h) only criticizing me when colleagues and I make mistakes together
Undermining	Social alienation	(10a) treating me as negative example when he/she talks to others
		(10b) using irony to make me embarrassed and look bad in the eyes of others
		(10c) speaking ill of me or giving me a negative review in my absence
		(10d) instigating colleagues not to have too much contact with me
	Work-related disapproval	(11a) suggesting that I have little developmental potential
		(11b) suggesting that I do non-value work
		(11c) questioning my personal qualities and work capabilities
		(11d) assigning me to an unimportant position without any explanation

**Table 2:** Coding categories and definitions of the subcategories.

Subcategories	Description
Interpersonal ignoring	Treating the target as a non-existing group member by giving no attention when attention could be reasonably expected during the course of daily communication and contact.
Work-related ignoring	Failing to provide the target with due attention in relation to performance, progress and contributions.
Emotional neglect	Failing to offer emotional support when the situation calls for it.
Work-related neglect	Overlooking and/or hindering the target from having legitimate needs and expectations met in relation to work.
Prevarication	Responding in an evasive manner when help or support is needed.
Social exclusion	Keeping the target on the outside of the social fellowship in the workplace.
Work-related exclusion	Failing to include the target in work-related activities where inclusion is expected.
“Insider’s” favoritism	Giving other employees better treatment to an illegitimate degree, compared with the target.
“Outsider’s” derogation	Giving the target worse treatment to an illegitimate degree, compared with other employees.
Work-related disapproval	Failing to give due recognition to the target’s ability and potential at work.
Social alienation	Negatively affecting the social status and position of the target.

### General Ignoring

Altogether ten respondents reported behaviors that could be grouped together under the core category “general ignoring,” which mostly occurred during daily interactions and work-related contact with leaders. Furthermore, as shown in Table 1, two sub-categories emerged based on the experiences of the participants, i.e., *interpersonal ignoring* (e.g., “not responding to my warm greeting”) and *work-related ignoring* (e.g., “passing me by when everyone has a chance to express opinions in meetings or discussions”). For example, one respondent who reported interpersonal ignoring said the following:

“I found my supervisor was unwilling to chat with me if the conversation was not necessary. For instance, she seldom talked to me in a group chat. Sometimes, she even chose to walk away to avoid talking to me when I tried to join her conversation with other colleagues.”

Another respondent reported being ignored in relation to work, and said the following:

“I thought some of my progress was obvious enough to be noticed. Nevertheless, my manager always gave me a cold shoulder, no matter how big my efforts and progress.”

These quotes highlight instances where leaders either keep distance or turn a blind eye to the targets’ existence in both daily communication and with respect to performance at work, in a manner that appears to convey that the subordinate does not exist or does not belong there. General ignoring is hence characterized by typical acts of omission where leaders avoid giving due care and attention when responses and attention generally would have been expected. Importantly, most of the ten respondents exposed to general ignoring behavior emphasized that they would not define the behavior as such when first exposed. In fact, most targets were reluctant to accept and face that fact until repeatedly having been ignored by their leader. For instance, one respondent stated:

“When I first encountered a situation where my leader made no response to my greeting and messages, I would find many excuses for him. For example, if he did not respond to my greeting it might be because the distance between us is too great; he did not reply to my message because the SMS was flooded by others (…). But after many similar situations, I was sad when I actually realized the fact is that my manager doesn’t want to have much personal contact with me.”

Overall, “general ignoring” seemed to emerge as a rather common theme in experiences of leadership ostracism in the present sample. Taking all of the reported examples into account, general ignoring can take an interactional or a work-related form, and can be described as behaviors by which an immediate leader treats a subordinate as if he or she does not exist at the workplace. For an overview of all included acts of ignoring, see Table 1.

### Neglect

Twelve respondents reported a type of leadership ostracism behavior that could be grouped together under the core category “neglect.” These acts reflect a type of ostracism behavior that occurs when leaders specifically ignore particular and specific employee needs or when the situation otherwise entails a concrete expectation about being given attention. Furthermore, below the label “neglect,” three separate sub-categories emerged from the data, comprising *emotional neglect* (e.g., “not comforting me when I feel discouraged encountering obstacles or difficulties”), *work-related neglect* (e.g., “not introducing me when I should be introduced”) and *prevarication* (e.g., “finding some excuse to refuse me when I ask for help”).

According to the reported examples, we found that Chinese employees often believe that supervisors to a certain degree have a general duty of care, involving responsibility for providing legitimate care and emotional support. However, Chinese leaders do not generally regard this responsibility as a necessary and official duty. Thus, any threat to the employees’ fundamental need to belong represented in terms of neglect is reinforced by the respondents’ belief that leaders choose to offer less care and attention when they feel a lower degree of social attraction toward them. For example, one respondent reported an experience of emotional neglect as follows:

“I called my dean to tell him that we lost the bid of a project where we had put in serious and sustained effort, because I thought he would be the one who would really understand my disappointment. His response with a simple sentence saying ‘do you have anything else to report?’ did not ease me, but rather made me sink into self-blame.”

Another respondent who was subjected to work-related neglect reported the following experience:

“I left my last job because I was swamped with work. I had kept requesting my manager to provide me with a new assistant since 3 months before my present assistant left, as I realized she was going to take maternity leave. Although the manager said okay every time, he never dealt with it. It seemed he never even took my request into account.”

Additionally, in some cases, leaders’ seemingly positive answers or a euphemistical excuse could serve as a mask for their rejection, in order to cover up neglect that might be purposeful. For instance, one respondent employed at an intermediate agency for studying abroad^1^ reported prevarication in the following way:

“In my organization, taking a delegation of students to the United States is a kind of reward for employees who can achieve a challenging performance goal. Although I have exceeded my goals and been qualified two times during my three and a half year tenure in this company, I never got any opportunities to go to America. Each time I asked my manager whether I could lead the students to the United States, I was told ‘You have my words—I will let you go next time’.”

Overall, participants’ statements show that “neglect” is also a quite common dimension of leadership ostracism behavior in the present sample. Taking all of the reported examples into account, “neglect” can take the form of emotional neglect, work-related neglect and prevarication, and may be described as behavior where an immediate leader is oblivious to the reasonable expectations and needs of a subordinate at the workplace. For an overview of all included acts of neglect, see Table 1.

### Exclusion

Seven respondents reported a type of leadership ostracism behavior that we grouped and classified as “exclusion.” Furthermore, as shown in Table 1, two sub-categories emerged in the form of *social exclusion* (e.g., “inviting other colleagues to join his/ her after-work gathering, but not me”) and *work-related exclusion* [e.g., “not bringing work-related activities to my attention (like important meetings or business trips)”]. For example, one respondent who reported experiences with social exclusion said:

“I sometimes see my group leader and other group members leave together after work. I didn’t give it much thought until once I saw one of them share pictures taken at her (the leader’s) birthday party in her home. I realized I was excluded by my group leader as I was one of the only two members who were not invited.”

As an example of work-related exclusion, another respondent said:

“On the first workday of this year, my manager asked one employee to inform the other colleagues that there would be a meeting in his office. Except me. One of them came out and told me they had checked the work plan for the new year. I felt upset, that I was in a dangerous situation, as my manager excluded me from such an important meeting.”

Based on the statements of our respondents, “exclusion” denotes actions or inactions where employees are not included in activities where inclusion would be expected. Specifically, “exclusion” can be fundamentally social or work-related, and can be described as behavior by which an immediate leader directly or indirectly keeps a subordinate outside of the social or professional fellowship in the workplace. For an overview of all included acts of exclusion, see Table 1.

### Differential Treatment

Altogether fifteen respondents reported a type of leadership ostracism behavior denoting unfair and differential treatment as compared with the treatment of other colleagues. We have grouped these behaviors together under the term “differential treatment.” Furthermore, as shown in Table 1, two subcategories were identified, i.e., *“insider’s” favoritism* (e.g., “preferring my colleagues over me for certain positions that can generate immediate benefits”) and *“outsider’s” derogation* (e.g., “providing me with less convenience at work than other colleagues”). As an example of insider’s favoritism, one respondent said:

“No matter how much effort I have made to establish a good relationship with my leader, I sense I am an outsider with no acceptance. He chose to trust certain other colleagues and shared his thoughts about work and life with them, but barely said anything to me.”

As an example of “outsider’s” derogation, another respondent said:

“I was one of the project leaders in my department, but was in charge of a project with less funding than some of the other projects. Although we are all project leaders in the same level of the department, I got less resources and support by my leader. In the case where all my team members including me were using old computers with poorer configuration, my leader still assigned new computers to another group. As such, our efficiency was not as good as that of others. My leader then asked me to hand over some of our work to other project teams. I feel my leader was pushing me out by carving up my project.”

As evident here, differential treatment as a type of leadership ostracism is rooted in psychological comparison and a resulting sense of psychological imbalance. Importantly, insider’s favoritism can occur without any harmful leader behavior directed at the target, also comprising behavior that merely benefits other colleagues in a way that is seen as illegitimate or as an implicit sign that one is not included in the leader’s inner circle. With regard to the perception of being an “outsider,” interviewees describe leaders’ unjust treatment as a lack of attention and emotional support, and/or poorer access to organizational resources by virtue of comparison with the treatment of their co-workers.

Importantly, such “insider” versus “outsider” comparisons may be influenced by the typical Chinese “guanxi” orientation context, and may therefore be especially relevant in the present research context. Overall, “differential treatment” can be either insider-oriented or outsider-oriented, and can be described as behavior by which an immediate leader treats a subordinate as an unvalued member of the collegial fellowship at work relative to others. For an overview of all included acts of differential treatment, see Table 1.

### Undermining

Twelve respondents reported a type of leadership ostracism behavior that could be labeled as “undermining.” As a dimension or core category of leadership ostracism, undermining appears in our data to reflect acts that damage either the social or the professional position or identity of the target, thereby hindering inclusion and belongingness. Several respondents phrased relevant experiences in the form of Chinese proverbs, like “dressing me in small shoes”^2^. Furthermore, as shown in Table 1, two sub-categories were established based on the list of relevant items, i.e., *social alienation* (e.g., “instigating colleagues not to have too much contact with me”) and *work-related disapproval* (e.g., “giving me lower performance rates than others who perform similar to me”), reflecting threats to social and professional status (including career development), respectively. As an example of social alienation, one respondent said:

“My team leader used humorous but ironic words to make jokes about me to a new employee in the workplace. Even if it was a joke, it made me embarrassed as others who did not recognize that fact could think I was the one always making mistakes in the group.”

As an example of work-related disapproval, another respondent said:

“No matter how much effort and progress I did and gained, my manager seemed not to recognize my ability by questioning whether I had finished the tasks myself, without help from others.”

Taking all of the reported examples into account, “undermining” can take the form of social alienation and work-related disapproval, and can be described as behavior by which an immediate leader belittles an employee’s ability and potential in the job and negatively manipulates his or her social position at work. For an overview of all included acts of undermining, see Table 1.

## Discussion

The purpose of this study was to explore the relevant behaviors associated with the concept of leadership ostracism in a Chinese work context by investigating how subordinates perceive ostracism as carried out by their immediate supervisor. Previous studies on ostracism behavior in the work context have tended to focus on antecedents and outcomes from the perspective of the targets. In this study, we have rather focused on the content and behavioral typology of leadership ostracism in itself, specifically looking at the behavioral content of the construct. Thus, the present study is one of the first to address the very nature and characteristics of this particular form of workplace ostracism, and demonstrates that it may be described in terms of five dimensions, based on in-depth interviews with Chinese employees (see Figure 1). Specifically, the dimensions, or core-categories, of leadership ostracism as it emerges from the perceptions of Chinese workers, comprise *general ignoring, neglect, exclusion, differential treatment*, and *undermining*. We posit that these dimensions denote different forms of leadership ostracism that alone or in combination act as sources of deteriorated belongingness and inclusion at work. Hence, we suggest that leadership ostracism may be described as actions or inactions by a leader that in the form of general ignoring, neglecting, exclusion, differential treatment and/or social undermining negatively affects the social position, professional position, career development, inclusion and/or the quality of the interpersonal workplace relationships of an employee.

This description resembles previously suggested definitions of workplace ostracism, such as that of Hitlan et al. (2006, p. 217), stating that workplace ostracism denotes acts of omission and open exclusionary behavior that “hinders one’s ability to establish or maintain positive interpersonal relationships, work-related success, or favorable reputation” at work. However, it also offers a more precise account of the behavioral aspects of ostracism apparent when enacted by an immediate leader. Thus, the present research is in line with Zadro and Gonsalkorale’s (2014) call to investigate and describe how the source of workplace ostracism itself may affect its behavioral manifestations.

Indeed, as shown in the present study, certain ostracism behaviors appear to be leader-specific [e.g., “(8c) preferring my colleagues for certain positions that can generate immediate benefits”], while others yet earn their strength and impact from the leader position [e.g., “(5b) finding some excuse to refuse me when I ask him/her for help”]. Thus, although ostracism as carried out by a leader conceptually overlaps with workplace ostracism enacted by other organizational members, their behavioral expressions are not necessarily the same according to our results.

Additionally, the present study identifies certain types of ostracism behaviors not traditionally described as such. For example, the sub-category *social alienation* (a form of undermining) comprise acts that manipulate the social position and status of an employee in a manner that hampers inclusion and belongingness [e.g., “(11f) instigating colleagues not to have too much contact with me”]. Thus, the present study also suggests an expanded view on the behavioral typology of ostracism, thereby further adding to its novelty and contribution. In the following, we discuss each core category in greater detail.

### General Ignoring

The first core category emerging from the interviews was “general ignoring.” The term “ignoring” has frequently been used to describe ostracism in the literature (e.g., Williams et al., 2013; Zadro and Gonsalkorale, 2014), and is posited to denote a key characteristic of ostracism in any form (Williams, 2007). Based on our interview data, we understand leadership ostracism in the form of general ignoring as “an immediate leader treating a subordinate as if he or she does not exist at the workplace.” This implies that general ignoring largely comprise omissions to involve or relate to the target on a general basis, rather than enacting neglect in response to a situation that specifically calls for attentiveness and responsiveness.

We were also able to differentiate between two sub-dimensions of this core category, namely interpersonal and work-related general ignoring. *Interpersonal ignoring* refers to acts or omissions that implicitly disregards the existence of an individual during the course of interpersonal interactions. As such, interpersonal ignoring may lead to an experience of being worthless and disliked as a fellow human being, attributions held to be common among ostracism targets (Zadro et al., 2004). *Work-related ignoring*, on the other hand, refers to acts or omissions that implicitly convey professional redundancy, involving a disregard of the worth of one’s contributions at work. Hence, while a qualitative distinction between these sub-dimensions is apparent, they also share a conceptual base, conveying that the target is worthless and unimportant enough not to be noticed. Repeated experiences of general ignoring may thereby reflect an implicit and often subtle threat to belongingness, self-esteem, meaningfulness and control (Williams, 2009).

### Neglect

The second core category emerging from the data was “neglect.” As with general ignoring, neglect has previously been established as a form of ostracism and is evident in existing workplace ostracism scales. For instance, from the workplace ostracism scale of Hitlan and Noel (2009), the item “Supervisors not replying to your requests/questions within a reasonable period of time” appears to fully overlap with neglect as a dimension of leadership ostracism in the present study. Moreover, in the Bullying Ostracism Screening Scale (BOSS) (Gilman et al., 2013), “socially neglected” is defined as one of the behavioral subtypes, also indicating that neglect denotes a form of leadership ostracism. At the same time, we went through several discussions about this specific core category and its relation to general ignoring, as the two may appear to be highly overlapping, and as the translation of ignoring and neglect into Chinese are very similar [i.e., “
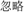
”(hū lüè) and “
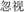
”(hū shì)]. However, as there is a fundamental difference between the two in terms of whether it is the employee him/herself or his/her needs and expectations that is disregarded, we hold that general ignoring and neglect can be reasonably distinguished from one another. Specifically, while general ignoring entails acts or omissions that disregard the existence of an employee *per se*, neglect entails a disregard of legitimate and more or less obvious needs and expectations presenting themselves during the workday.

Interestingly, this particular form of leadership ostracism highly resembles so-called laissez-faire leadership, an established form of destructive leadership wherein the supervisor fails to respond to the needs of his or her subordinates (Bass, 1990; Skogstad et al., 2007). In fact, scholars have previously argued for a potential link between laissez-faire leadership and ostracism, holding that the two may overlap (Skogstad et al., 2014). Seeing as laissez-faire leadership denotes a harmful and destructive leadership style with detrimental outcomes, particularly when job demands are high, neglect as a form of leadership ostracism may also potentially prove to be a significant source of subordinate distress.

The core-category neglect may, according to our findings, be divided into three sub-categories. *Emotional neglect* refers to acts or omissions reflecting a leader’s obliviousness to the needs and expectations of an employee when facing particular emotional challenges. *Work-related neglect*, which comprised the largest sub-category in this dimension in terms of the number of items (see Table 1), refers to acts or omissions reflecting a leader’s obliviousness to the needs and expectations of an employee in relation to challenges, efforts and/or progress at work. This may include non-responsiveness when problems arise at work and when the employee could reasonably expect help and support from his/her nearest leader, potentially with the effect of hindering the employee’s work-related success. Finally, the sub-category *prevarication* emerged as a type of leader behavior appearing to reflect an ambiguous form of rejection where leaders relate to the subordinate’s needs in an evasive manner. Possibly, the relevance of this sub-category should be interpreted with regard to Chinese cultural values, and the common tendency to avoid direct confrontation in order to maintain “face” and to appear moderate. Importantly, the inherent ambiguity of this neglectful treatment is likely to make targets sink into self-doubt, and to experience emotional distress, anxiety and anger (Twenge et al., 2001; Lustenberger and Williams, 2009) by virtue of entailing a significant threat to the belongingness of an employee.

### Exclusion

The third core category emerging from the data was “exclusion.” Generally, exclusion is held as yet a central and defining feature of ostracism, and has even been used as a synonym for ostracism in previous work within social psychology (e.g., Eisenberger et al., 2003; Abrams et al., 2004; Hitlan et al., 2006; Williams et al., 2013). As such, we were somewhat surprised to observe that relatively few interviewees (*N* = 7) had experienced exclusion from their supervisor. However, it is possible that exclusion could prove to be more prevalent when co-workers enact workplace ostracism, as it often involves exclusion from a group or an entire fellowship (Williams, 2007). Moreover, as outright exclusionary acts may be regarded as rather overt in the present research context, it is also possible that Chinese leaders are prone to more subtle and ambiguous behaviors when ostracizing their subordinates. Hence, we believe this finding to be influenced by the deeply rooted “moderation” of Confucianism and cultural importance of maintaining “face.” We are, however, uncertain as to how prevalent exclusion would be in other cultural contexts when investigated as a dimension of leadership ostracism, and thus encourage other scholars to pay close attention to this finding in particular upon doing similar investigations in other cultures.

As a type of ostracism behavior played out by leaders, exclusion can be described as situations where an immediate leader directly or indirectly keeps a subordinate outside of the social and professional fellowship in the workplace. This description reflects both of the sub-dimensions established in our study, where *social exclusion* refers to exclusionary acts that keep the employee on the outside of the social fellowship, and *work-related exclusion* refers to behavior directly or indirectly failing to include the employee in work-related activities where inclusion would be expected. Together, these related forms of leadership ostracism may marginalize the employee and his/her social position, thwarting fundamental social needs in the work context (Williams and Zadro, 2001).

### Differential Treatment

The fourth core category emerging from the interviews was “differential treatment,” which denotes treatment that is perceived as unfair as the leader distributes organizational resources and emotional support in an unequal and socially illegitimate manner, implying that certain subordinates are closer and more central to the leader than are the targets. While differential treatment in itself is often linked with injustice perceptions in organizational studies (e.g., Van Breukelen et al., 2002), it is our position that it may also comprise a form of ostracism to the degree that it violates a psychological contract concerning one’s inclusion and social value. For this reason, differential treatment may be particularly relevant for the perception of the “guanxi” structure in the Chinese cultural context. Specifically, in Chinese working life, an unspoken psychological contract often exists between the leader and the subordinates, including an agreement about the closeness and intimacy of their relationship. Hence, when leaders violate this contract by treating an employee as if more interpersonally distal than expectations and unspoken agreements stipulate, the treatment denotes a form of rejection, which comprise a significant threat to inclusion and belongingness. Importantly, this means that treating a subordinate differently relative to other employees does not imply ostracism in and of itself, but may do so under conditions of a given “guanxi” structure. In this respect, differential treatment denotes a form of leadership ostracism to the degree that a leader uses his or her position to force the subordinate out of the warmth of the inner circles by treating them as if they have less of a place than they expect.

The two sub-categories described under this core category reflects two sides of the same coin. Specifically, “outsider’s derogation” refers to ostracism where a leader treats a subordinate as a member of distal “guanxi” circles in a socially illegitimate manner. Conversely, as a less direct form of ostracism, “insider’s favoritism” may represent an inclusion threat, as the leader treats other members of the collegial fellowship as members of central circles in a manner that is perceived as unjust relative to the perceived “guanxi” position of the ostracized employee. This notion is in line with research on the indigenous Chinese leader member guanxi (LMG), evolved from the leader-member exchange theory (LMX), where out-group members are shown to regard themselves as targets of ostracism when experiencing less inclusion, affective attachment, help and support from their leader (Graen and Uhl-Bien, 1995; Ying et al., 2014).

Altogether, the core category *differential treatment* entails leader behavior by which a subordinate is treated as an unvalued member of the collegial fellowship at work relative to others with respect to the prevailing psychological contract. As a form of leadership ostracism, differential treatment thereby poses a significant threat to inclusion and belongingness at work.

### Undermining

The fifth core category emerging from the interviews was “undermining.” In the present study, we understand leadership ostracism in the form of undermining as an immediate leader negatively manipulating a subordinate’s social position and/or belittling his or her work contributions and potential in the job. Furthermore, two sub-dimensions of undermining emerged from the data, i.e., social alienation and work-related disapproval. *Social alienation* appears in our data as undermining behavior enacted by a leader in order to negatively affect the social position of the target, which may be significantly related to ostracism as it diminishes the target’s degree of inclusion and belongingness. Examples such as “speaking ill of me or giving me a negative review in my absence,” implies that the said employee is of less worth to the group, eligible to be embarrassed and belittled in front of other group members. This treatment may also hinder the target’s ability to maintain positive interpersonal relationships or favorable reputations in the workplace, in line with the workplace ostracism definition of Hitlan et al. (2006). Hence, this finding comprises an important addition to the existing literature on workplace ostracism.

The second sub-dimension of undermining, *work-related disapproval*, refers to acts perceived by the targets as devaluations of their working ability, job value and potential at work, again with relevance to Hitlan et al.’s (2006) notion concerning targets’ ability to establish and maintain work-related success. As a form of leadership ostracism, this behavior can be rather ambiguous, possibly disguised as legitimate critique, and may exert a strong threat to targets’ self-esteem, sense of control and one-to-one inclusion, thus reflecting a final sub-dimension of leadership ostracism, as it emerges from the data of the present study.

## General Discussion, Implications, and Conclusion

The present study is conducted from a target perspective in a distinct cultural context. As such, culture-specific social norms may have influenced the results. Although we hold that this setting is well suited for exploring leadership ostracism, the degree of generalizability to other cultural settings remains unaccounted for. For example, it would be interesting to see whether employees in other cultures consider differential treatment as ostracism. We would also be interested to see whether social alienation can be established as a form of leadership ostracism elsewhere, and would welcome any attempt to replicate our findings in other research contexts. In addition, we note that although we attempted to reduce the analysts’ subjectivity as far as we could, they were not eliminated. Our subjectivity may, for example, have affected the study’s results when we selected and coded first-order labels across the textual data based on our understanding of leadership ostracism. On the other hand, theory emerges not solely from the reported experiences of the participants but in concert with researchers’ observations and subjective understanding (Charmaz, 2006; Alrøe and Noe, 2014). Moreover, the use of analysts with different cultural and scholarly backgrounds should have prevented any undue impact of the researchers’ subjectivity.

Additionally, as China is presently experiencing an economic downtrend, the present research could be vulnerable to influence at a national level. For example, while employment rates have gone up for some years, the more recent negative economical trend in China has led to downsizing, possibly increasing general levels of job insecurity, making employees even more attentive to any negative behavior from their leaders and affecting levels of work-related well-being (Burns et al., 2012; Giorgi et al., 2015; Mucci et al., 2016).

In terms of contributions, the present study’s focus on how ostracism is enacted by leaders represents a novel approach to the documentation of ostracism in working life. Specifically, while leadership ostracism has previously been treated as fairly equivalent to workplace ostracism in general, we have sought to describe the subjective experience of leadership ostracism from the targets’ perspective in an inductive manner. The resulting behavioral model reflects a wide array of acts. Many of these partly or fully overlaps with acts traditionally associated with ostracism, such as social exclusion and interpersonal ignoring. However, the behavioral typology of leadership ostracism identified also include acts not typically seen in the ostracism literature, such as social alienation and work-related disapproval. Hence, while replicating several established notions, the present study also unveils a leader-specific range of tactics and behaviors relating to ostracism, at least as perceived and construed by the targeted subordinates. Partly, these behaviors may be unique for the leader role, and partly they gain added impact from that role. This fact alone should render the content and behavioral typology model put forward here a significant starting point for further research on the topic. For instance, the items of leadership ostracism identified in the present study may represent a foundation for the development of a questionnaire to be used in quantitative research with larger samples.

From a practical perspective, the present study shows that leadership ostracism is not only unpleasant, but can negatively affect subordinates’ career development, social position and personal reputation at work. Hence, in some respects, leadership ostracism entails both a different and possibly greater threat to employee well-being than acts of ostracism carried out by co-workers or subordinates. For instance, the differential treatment seen in some of the categories directly threatens interactional justice at work, where less opportunities and resources in the form of benefits, training, and promotions are given to targets of leadership ostracism (Stamper and Masterson, 2002). It may also deplete employees’ psychological resources by consuming time, energy and efforts needed to move back toward to the inner circles of leader “guanxi.” Additionally, acts of social alienation may signal a leader’s aversiveness, can yield strong feelings of estrangement and possibly induce group isolation due to a trickle-down effect of leadership behavior and attitudes (Mawritz et al., 2012).

In conclusion, the present study demonstrates that leadership ostracism can take several forms, some of which include behaviors not traditionally associated with ostracism in the workplace. Specifically, using in-depth interviews with Chinese employees and analyses based on grounded theory, we have shown that leadership ostracism may take the form of general ignoring, neglect, exclusion, differential treatment and undermining. We believe that the present study may hold important practical implications, and that it may serve as a springboard for future empirical research on leadership ostracism, preferably across different cultural contexts. We also encourage future studies on where, when, and how leadership ostracism is most likely to occur in organizations, and how ostracized employees may react to such treatment.

## Author Contributions

ZC contributed to conception and design of the study. MZ organized the interview and collected the text data. All authors contributed to the data analysis. MZ and MG contributed to the first and subsequent drafts of the manuscript. All authors contributed to manuscript revision, read and approved the submitted version.

## Conflict of Interest Statement

The authors declare that the research was conducted in the absence of any commercial or financial relationships that could be construed as a potential conflict of interest.

## Footnotes

1 With hundreds of thousands of Chinese students aiming for an overseas education around the world every year, study abroad agencies, which normally offer overseas study services, have become a fast-growing and lucrative sector in China.

2 “Dressing me in small shoes” is a well-known daily language, which means making things hard for someone.

## Appendix

### Interview Protocol

(1) Have you ever heard of leadership ostracism?
(2) Could you talk about how you understood leadership ostracism when you first heard about it?
(3) Based on your description of leadership ostracism, have you ever been exposed to such treatment?
(4) Could you describe your feelings and the situation when exposed to ostracism from your leader?
(5) Are your co-workers also treated with ostracism behavior from your leader? If not, how does your leader treat others?
(6) Besides your own experience, have you ever seen or heard that colleagues or friends say they have experienced leadership ostracism? Please give some examples.
(7) What may be the reason that your colleagues and friends are ostracized by their leaders?
(8) How would you classify the leadership ostracism acts you have mentioned?
